# Bullous Skin Diseases: Classical Types of Autoimmune Diseases

**DOI:** 10.1155/2013/457982

**Published:** 2013-01-08

**Authors:** Jan Damoiseaux

**Affiliations:** Laboratory of Clinical Immunology, Maastricht University Medical Center, P. Debyelaan 25, 6229 HX Maastricht, The Netherlands

## Abstract

The prototypic bullous skin diseases, pemphigus vulgaris, pemphigus foliaceus, and bullous pemphigoid, are characterized by the blister formation in the skin and/or oral mucosa in combination with circulating and deposited autoantibodies reactive with (hemi)desmosomes. Koch's postulates, adapted for autoimmune diseases, were applied on these skin diseases. It appears that all adapted Koch's postulates are fulfilled, and, therefore, these bullous skin diseases are to be considered classical autoimmune diseases within the wide and expanding spectrum of autoimmune diseases.

## 1. Introduction

The bullous skin diseases, including pemphigus and bullous pemphigoid, affect the skin and/or oral mucosa. Since the skin is a vital organ in the protection of the body against dehydration and infections, these skin diseases may be life threatening. The bullous skin diseases are being divided in two categories based on whether the skin is affected within the epidermis or at the epidermal-dermal interphase. The first category is referred to as pemphigus and entails 4 disease entities: pemphigus vulgaris, pemphigus foliaceus, paraneoplastic pemphigus, and IgA pemphigus. Altogether, the yearly incidence of this category is about 0.3/100,000 and the age of onset of these diseases is primarily in the fifties and sixties. The second category entails multiple disease entities. When considering dermatitis herpetiformis, a bullous skin manifestation of celiac disease, as a distinct subcategory of the pemphigoid skin diseases, the overall yearly incidence of the second category is about 1.0/100,000. These diseases typically become manifest at an age >65 years [1–3]. 

The diagnosis of the bullous skin diseases is based on the typical skin manifestations, which may be objectified by the Nikolsky sign and characteristic direct immunofluorescence (DIF) patterns in skin biopsies (Table 1). The presence of skin-specific autoantibodies in the circulation will further add to the diagnosis [1].

In the current paper Koch's postulates for defining infectious diseases and adapted for autoimmune diseases are applied on both categories of bullous skin diseases [4, 5]. Since these skin diseases fulfil all criteria, they can be considered to belong to the small number of unequivocal autoimmune diseases within the ever expanding number of diseases that are supposed to be autoimmune.

## 2. Koch's Postulates Adapted for Autoimmune Diseases

While Koch's postulates were originally intended to define the infectious origin of a disease, they have been adapted for demonstrating that diseases are due to the loss of self-tolerance, that is, autoimmune diseases [4, 5]. The first postulate is the demonstration that the adaptive immune system is directed to self-components present in the affected tissue. The second postulate requires that this autoreactivity is also localized within the affected tissue. For both the first and the second postulate, the autoreactivity of the adaptive immune system may comprise antibodies and/or T cells. Antibodies can be easily detected in the circulation by many different immuno-assays [6], but T-cell autoreactivity requires individualized assays because of the MHC-restriction of antigen recognition. Within the tissues, the localization of autoantibodies becomes manifest by DIF. For T cells, immunohistology is required to reveal their presence in the tissue but it is hard to distinguish self-reactivity from bystander infiltration.

The third postulate states that the transfer of the autoreactive components from a patient to a healthy individual should induce similar disease manifestations in the recipient as observed in the donor. Obviously, in case of autoimmune diseases this is an unethical issue and therefore the recipient may also be an animal to meet this criterion. However, especially in case of autoantibodies, Mother Nature provides a situation where this criterion is also fulfilled in the human situation. Indeed, during pregnancy antibodies are transferred from the mother to the unborn child. If the mother has pathogenic autoantibodies in the circulation, these will also be transferred via the placenta to the child and cause disease manifestations characteristic of the respective autoimmune disease. If so, the child will recover from the disease upon disappearance of the maternal antibodies. For T-cell mediated autoimmune diseases, again, it is difficult to meet the adapted postulates due to MHC restriction.

The fourth postulate concerns the induction of the autoimmune disease by immunization protocols, typically performed in animals. This requirement depends on the identification of the autoantigen, and if this is known, it can be met for both antibody and T-cell mediated autoimmune diseases. Furthermore, if immunization for a T-cell mediated autoimmune disease is effective, adoptive transfer of T cells from diseased animals to healthy recipient animals becomes feasible and this can be considered a surrogate for the third postulate. As we will see (vide infra), genetic modification of animals has enormously expanded the possibilities to induce autoimmune diseases in experimental animal models.

The last postulate is about therapeutic efficacy. Only therapy that directly targets the adaptive immune system, in particular the autoreactive component should result in amelioration or even cure of the disease. Preferentially, this should be accompanied by a reduction of the responsible autoimmune component.

## 3. Desmosomes and Hemidesmosomes

The barrier function of the epidermis requires that the epithelial cells are closely associated to each other, as well as to the basement membrane. The intercellular adhesion involves the desmosomes, while the interaction of the basal epithelial cells with the basement membrane is fulfilled by hemidesmosomes. In desmosomes, the intercellular interaction is elaborated by a heterotypic binding of two membrane-bound adhesion molecules, desmoglein (Dsg) and desmocollin [7]. Both molecules are intracellularly linked to the cytoskeleton via intracellular plakoglobin, plakophilin, and desmoplakin. Interestingly, Dsg entails two subtypes, Dsg1 and Dsg3, which are differentially expressed in the skin and oral mucosa [8]. In the skin, Dsg3 is only expressed in the basal layers of the epithelium, while Dsg1 is strongly expressed in the outer layers with decreasing expression levels towards the basal membrane. In the oral mucosa, however, Dsg3 is strongly expressed homogenously by all epithelial cells. The expression of Dsg1 reveals a similar gradient as in the skin, but expression levels in the oral mucosa are much lower than in the skin. This implies that epithelial integrity in the oral mucosa is almost exclusively established by Dsg3, while in the skin Dsg3 and Dsg1 are primarily responsible for the integrity of the basal and outer layers of the epithelium, respectively. 

Binding of the basal epithelial cells to the basement membrane is effectuated by the membrane-bound BP180, also known as collagen type 17. This interaction is further supported by the *α*6*β*4 integrin, binding laminins in the basement membrane, and the tetraspanin CD151. Both the integrin as well as BP180 are intracellularly linked to the cytoskeleton via BP230, also referred to as bullous pemphigoid antigen 1 (BPAG1e) and plectin [8]. The basement membrane consists of three layers: the lamina lucida (epidermal site), the lamina densa (middle), and the fibroreticular lamina (dermal site). All these layers consist of extracellular matrix components, like fibrinogen and collagen types 1 and 7. Importantly, the interaction between the lamina lucida and lamina densa is based on chaotropic binding. The incubation of skin tissue with high salt (1 M NaCl) results in loss of integrity (salt-split-skin) between both layers resulting in the lamina lucida being attached to the epidermal part (roof) and the lamina densa to the dermal part (bottom). Obviously, lamina components will be differentially distributed between both parts.

## 4. Adapted Koch's Postulates Applied on Pemphigus

The two most representative entities of the pemphigus diseases are pemphigus vulgaris and pemphigus foliaceus [3]. While pemphigus vulgaris is primarily a blistering disease of the oral mucosa, pemphigus foliaceus is exclusively a blistering disease of the skin. Some patients with pemphigus vulgaris, however, show also bullous skin manifestations. These diseases are characterized by the differential presence of autoantibodies to the desmosome components Dsg1 and Dsg3 [9]. In pemphigus vulgaris circulating IgG antibodies to Dsg3 are present either alone or in combination with antibodies to Dsg1. In pemphigus foliaceus, however, only IgG antibodies to Dsg1 are detectable in the circulation. These antibodies can be detected by indirect immunofluorescence (IIF) assays on preparations of the distal part of the oesophagus. In this assay, the antibodies reveal a typical chicken-wire staining pattern of the intercellular components in the epidermis. Next, antigen specificity can be demonstrated by enzyme-linked immunosorbent assays (ELISA) using Dsg1 and Dsg3 as antigens. These autoantibodies are not only present in the circulation, but also in the affected tissues as detected by DIF in tissue biopsies. DIF reveals a similar intercellular staining pattern of the epidermis as observed in the IIF. Additionally, DIF may reveal deposition of complement at the sites where autoantibodies have bound to the tissues. The differential presence of autoantibodies, in combination with the differential expression of the autoantigens explains the distinct disease manifestations of pemphigus vulgaris and foliaceus, that is, only superficial skin blistering in pemphigus foliaceus versus blistering within the oral mucosa with or without more basal skin blistering in pemphigus vulgaris [8]. The intraepidermal loss of integrity is also responsible for the classical Nikolsky sign. This is the phenomenon that upon lateral pressure on apparently healthy skin tissue the upper layers of the epithelium become loose.

Pathogenicity of the autoantibodies to Dsg1 and Dsg3 is to be expected because of the potential to disrupt desmosomal interactions. This is best demonstrated by the transfer of the autoimmune component. Although the age of onset of pemphigus is beyond the gestational age, cases of neonatal pemphigus have been described. This concerns both pemphigus vulgaris as well as pemphigus foliaceus [10, 11]. Both disease entities present in the neonate similarly as in classical patients. The neonates, however, do not produce the autoantibodies themselves and therefore they spontaneously recover upon resolution of the maternal antibodies. Due to similarity between human and murine Dsg proteins, passive transfer of autoantibodies to (neonatal) mice also results in the typical clinical manifestations of pemphigus [12], further demonstrating the pathogenic potential of these antibodies. The disease can also be adoptively transferred with patient lymphocytes to severely combined immunodeficient (SCID) mice [13]. However, the disease becomes only manifest in human skin transplants and not in the murine skin. Immunization protocols in animal models, however, are hampered by the strong tolerance for self-Dsg1 and self-Dsg3 in mice. To circumvent this problem, Dsg3-deficient mice were immunized with murine Dsg3. Next, spleen cells from these immunized mice were adoptively transferred to recombinase activating gene (RAG)2-deficient mice. Upon recognition of the Dsg3 in the recipient, the transferred Dsg3-specific lymphocytes induced lesions typical for pemphigus vulgaris: deposition of autoantibodies in the epidermis of the oral mucosa in a chicken-wire staining pattern and blistering within the basal epithelial layers [14].

The treatment of pemphigus is based on the suppression of the immune response and in particular the removal or neutralization of the autoantibodies. Depending on the severity of the disease different combinations of plasmapheresis, high dose intravenous immunoglobulins (IVIG), corticosteroids, rituximab, and cyclophosphamide are being used [15]. Because of toxicity, the latter is later on replaced by azathioprine or mycophenolate mofetil. Importantly, the resolution of the clinical manifestations and the normalization of the disease activity index scores are paralleled by the disappearance of the autoantibodies [9, 16].

## 5. Adapted Koch's Postulates Applied on Pemphigoid

Bullous pemphigoid is probably the most prevalent pemphigoid disease [3]. The characteristic subepidermal blistering reveals tense bullae. Since the epidermal-basement membrane integrity in the tissue neighbouring the bullae is also disturbed, finger pressure on the bulla will result in spreading of the blister. This is indicated as the alternative Nikolsky sign, originally referred to as the Asboe-Hansen sign. In pemphigoid, autoantibodies are directed to hemidesmosomal structural proteins, in particular, BP180. Like anti-Dsg antibodies, circulating IgG anti-BP180 antibodies can also be detected by IIF on sections of the distal part of oesophagus tissue. Anti-BP180 antibodies reveal a linear staining of the basement membrane zone. Since the BP180 molecule is expressed within the cytoplasmic membrane of the basal keratinocytes and anchored in the lamina lucida of the basement membrane, anti-BP180 antibodies reveal a “roof” staining by IIF in salt-split-skin preparations. In contrast, antibodies to collagen type VII, characteristic for patients with epidermolysis bullosa acquisita, react with the “bottom”-part of the salt-split skin [17]. The salt-split skin is considered the most optimal substrate for detecting antibodies to the basement membrane zone [18]. The antigen-specificity of the autoantibodies for BP180 can be defined by immunoassays, like ELISA [19]. Although also antibodies to BP230 are considered diagnostic markers for pemphigoid diseases, the test characteristics are not optimal [19]. Furthermore, with respect to pathogenicity, anti-BP230 antibodies seem less important because the BP230 antigens are expressed only in the cytoplasm [7]. Autoantibodies in bullous pemphigoid are not only present in the circulation but also deposit along the basement membrane of the skin. In DIF of skin biopsies a linear staining is observed, indicating deposition of IgG and complement. It should be stressed, though, that anti-BP180 antibodies may be present in the circulation in the absence of clinical manifestations of BP. Whether this situation represents a prodromal stage of BP, or is due to the overall increased prevalence of autoantibodies in the elderly, is an ongoing debate [20, 21].

Due to the very high age of onset of bullous pemphigoid, the occurrence of neonatal bullous pemphigoid is expected to be extremely rare and has, to my knowledge, not been described. However, another entity of the pemphigoid diseases, pemphigoid gestationis, is by definition associated with pregnancy and is also characterized by the presence of anti-BP180 antibodies. When pemphigoid gestationis becomes manifest during pregnancy, the autoantibodies will transfer the placenta and reveal typical skin lesions in the neonate [22]. Passive transfer of anti-BP180 antibodies to mice was not successful in inducing the disease and this has been attributed to molecular differences between human and murine BP180. The humanization of the autoantigen in mice, that is, using *Col17*-knockout mice rescued by the human ortholog, enabled the induction of characteristic skin lesions by passive transfer [23]. Immunization protocols in experimental animal models are enabled by the availability of genetically manipulated mouse strains. In case of bullous pemphigoid, wild type mice were grafted with skin of human COL17-transgenic mice. This induced an anti-human COL17 immune response in the engrafted wild type mice. Adoptive transfer of splenocytes from these immunized mice to RAG2-deficient, human COL17-transgenic recipients resulted in the production of anti-BP180 antibodies. In addition, the mice developed blisters and erosions corresponding to clinical, histological, and immunopathological features of bullous pemphigoid. Only eosinophil infiltration, one of the characteristic histological findings observed in patients, was not detected in the murine model [24].

The treatment of bullous pemphigoid is, like in pemphigus, based on immunosuppression in combination with elimination of the pathogenic autoantibodies. As shown in several case reports, disease activity scores are paralleled by concomitant shifts in the levels of autoantibodies, that is, disappearance of the autoantibodies when patients go in remission and increases in autoantibody levels at the time of a relapse [25].

## 6. Conclusion

Pemphigus vulgaris, pemphigus foliaceus, and bullous pemphigoid are three severe skin diseases that become clinically manifest predominantly in the elderly. The diagnosis is based on typical blister formation in the skin and/or oral mucosa, objectified by the Nikolsky sign and characteristic staining in both DIF and IIF. Within the wide and expanding spectrum of autoimmune diseases, these three bullous skin diseases are to be considered classical autoimmune diseases since they fulfil all of the adapted Koch's postulates.

## Figures and Tables

**Table 1:** Clinical and laboratory characteristics of bullous skin diseases.

Disease	Skin	Mucosa	Nikolsky sign*	DIF**	IIF	Autoantigen
Bullous pemphigoid	+	−	II	BMZ	BMZ	BP180
Pemphigus vulgaris	+/−	+	I	CWP	CWP	Dsg3 +/− Dsg1
Pemphigus foliaceus	+	−	I	CWP	CWP	Dsg1

*Nikolsky sign I is the classical Nikolsky sign; the alternative Nikolsky sign II was originally described as the Asboe-Hansen sign.

**BMZ: basement membrane zone; BP: bullous pemphigoid; CWP: chicken-wire pattern; DIF: direct immunofluorescence; Dsg: desmoglein; IIF: indirect immunofluorescence.
